# Inhibitory influence of natural flavonoids on human protein kinase CK2 isoforms: effect of the regulatory subunit

**DOI:** 10.1007/s11010-017-3228-1

**Published:** 2017-11-29

**Authors:** Andrea Baier, Jolanta Nazaruk, Anna Galicka, Ryszard Szyszka

**Affiliations:** 1 Department of Molecular Biology, The John Paul II Catholic University of Lublin, ul. Konstantynow 1i, 20-708 Lublin, Poland; 2 Department of Pharmacognosy, Medical University of Białystok, ul. Mickiewicza 2a, 15-089 Białystok, Poland; 3 Department of Medical Chemistry, Medical University of Białystok, ul. Mickiewicza 2a, 15-089 Białystok, Poland

**Keywords:** CK2 holoenzymes, Flavonoids, Phosphorylation, Inhibitors

## Abstract

CK2 is a pleiotropic, constitutively active protein kinase responsible for the phosphorylation of more than 300 physiological substrates. Typically, this enzyme is found in tetrameric form consisting of two regulatory subunits CK2β and two catalytic subunits CK2α or CK2α′. Several natural occurring flavonoids were tested for their ability to inhibit both CK2 holoenzymes, CK2α_2_β_2_ and CK2α′_2_β_2_. We identified few substances selectively inhibiting only the α′ subunit. Other compounds showed similar effect towards all four isoforms. In some cases, like chrysoeriol, pedalitin, apigenin, and luteolin, the α_2_β_2_ holoenzyme was at least six times better inhibited than the free α subunit. Otherwise, we have found a luteolin derivative decreased the kinase activity of CK2α′ with an IC_50_ value of 0.8 μM, but the holoenzyme only with 9.5 µM.

## Introduction

Protein kinase CK2 is a highly conserved serine/threonine kinase considered as constitutively active and ubiquitously expressed. CK2 is known to play an essential role in cellular processes, e.g., cell proliferation, cell growth, and cell survival, cell morphology, and promotes angiogenesis [1–9]. In many reports, the implication of CK2 in several pathologies is described, like neurodegenerative diseases (Parkinson’s and Alzheimer’s disease), inflammation, virus and parasite infections, and cancer [10].

CK2 can function as monomeric kinases, but also as tetrameric complexes. In human, the monomeric forms are designated as CK2α and CK2α′, being the catalytic subunits of CK2. The tetrameric isoforms consist of two catalytic subunits each bound to a regulatory subunit CK2β whereas two of them form a dimer. Within the CK2 holoenzyme, the regulatory CK2 subunit alters substrate specificity [11]. Calmodulin is only phosphorylated by the free catalytic subunits, while eIF2β is phosphorylated after reconstitution of the CK2 holoenzyme [12, 13]. The mechanism by which the regulatory subunit is able to regulate the activity of the catalytic subunit is still not clarified.

Both catalytic subunits possess very similar N-terminal amino acid sequences, whereas they differ at the C-terminal part. Characteristic features are the ATP-binding motif (G^46^XGXXS^51^), the catalytic loop (residues R^155^, D^156^ and H^160^), the activation loop (sequences D^175^WG^177^ and G^199^PE^201^), and the substrate binding site (residues R^191^, R^195^, K^198^, and K^74^-R^80^) [14, 15].

Beside these structural similarities distinct functional properties have been reported for both isoforms. As shown in mice, CK2α is expressed ubiquitously in all tissues, whereas CK2α′ is detected exclusively in the brain and in the testes [16]. Furthermore, knockout experiments in mice suggest discrete functions for these isoforms. Mice with knockout of the gene encoding for CK2α subunit are non-viable. Otherwise, mice lacking the CK2α′ subunit develop normally and survive into adulthood but males are infertile [17].

Since more than two decades research groups are in the search for potent and selective CK2 inhibitors. Those substances may act on several targets, like the ATP-binding site, the protein substrate-binding site, allosterically hindering substrate or co-substrate binding and inhibition of holoenzyme assembly [18]. The list of classes of compounds effectively inhibiting the CK2 activity is still growing, e.g., halogenated *1H*-benzimidazole derivatives, anthraquinones, coumarins, flavonoid derivatives, indeno[1,2-b]indole derivatives, pyrazolo-triazines, and carboxyl acid derivatives [19–25].

Recently, there has been an upsurge of interest in the therapeutic potential of medicinal plants which might be due to their phenolic compounds, specifically to flavonoids [26, 27]. They are most common and widely distributed group of compounds in plants, found in almost all plant parts, especially in photosynthesizing plant cells. They can be divided into a variety of classes such as flavones (e.g., flavone, apigenin, and luteolin), flavonols (e.g., quercetin, kaempferol, myricetin, and fisetin), flavanones (e.g., flavanone, hesperetin, and naringenin), and others. Being phytochemicals, flavonoids cannot be synthesized by humans and animals [28].

They have huge biological potential and promote human health helping to reduce the risk of diseases. Up to date about 9000 flavonoids have been identified [29].

In former studies, we detected significant differences between the inhibitory effects of halogenated *1H*-benzimidazole derivatives and flavonoid compounds towards both free catalytic subunits [20, 23]. The goal of our present study was to examine if this also holds true for the derived holoenzymes CK2α_2_β_2_ and CK2α′_2_β_2_. The regulatory subunit CK2β is known to alter the sensitivity of the CK2 molecule towards different substances, like NaCl and heparin.

## Experimental section

### Phytochemicals

Twenty-one compounds belonging to the various classes of flavonoids were isolated in the pure form from *Cirsium rivulare* inflorescences, *C. palustre* inflorescences and leaves, and *Erigeron acris* herb as described before [30–34]. All compounds were obtained from mentioned above plant sources after multistep chromatographic separations of methanolic extracts. The structure and purity of isolated compounds were elucidated on the basis of TLC, spectral analysis in UV and ^1^H and ^13^C NMR after comparison with compounds previously obtained in our laboratory, as well as comparison with literature data. Their purity was determined on the level at least 98%. Chrysoeriol was purchased from Roth (Germany).

### Purification of human CK2 holoenzymes

CK2α_2_β_2_ and CK2α′_2_β_2_ holoenzymes were purified as described elsewhere [35]. Briefly, both catalytic subunits were overexpressed as GST fusion proteins, whereas the regulatory subunit was expressed as His-tagged protein. *E. coli BL21(DE3)trxB* cells (Novagen) harboring the plasmid pGEX-3X::*CSNK2A1*, pGEX-3X::*CSNK2A2* or pET28a::*CSNK2B* were grown until OD_600_ = 0.6 at 37 °C. Next, IPTG was added to the final concentration of 0.2 mM; cultures were continued at room temperature for 4 h and then centrifuged at 5000×*g* for 10 min. Obtained bacterial pellets were mixed prior to lysis. Cells were disrupted by sonication and the supernatant was purified using glutathione-Sepharose (Pharmacia Biotech). Fractions containing the CK2 holoenzyme were pooled and dialysed against 50 mM Tris/HCl buffer pH 7.5 supplemented with 6 mM β-mercaptoethanol and 30% glycerol. The obtained protein preparations were used in enzymatic assays.

### Substrates

The protein substrate P2B used as phosphate acceptors was overexpressed and purified as previously described [23]. The acidic peptide with the sequence RRRADDSDDDDD was purchased from Sigma-Aldrich.

### Protein phosphorylation

CK2 activity was determined in a standard reaction mixture (40 μL of final volume) containing 20 mM Tris–HCl buffer, pH 7.5, 15 mM MgCl_2_, 6 mM β-mercaptoethanol, and 20 μM [γ-^32^P]ATP (specific radioactivity 500–1000 cpm/pmol) in the presence of 10 μM yeast P2B as substrate. Incubation was performed at 37 °C for 15 min. Afterwards, the reaction was terminated by adding 7 μL of SDS-PAGE loading buffer. Reaction mixtures were resolved in SDS-PAGE followed by Coomassie blue staining and autoradiography. The phosphate incorporation level in the P2B protein was estimated by cutting off the corresponding band and measuring the radioactivity in a scintillation counter (Perkin-Elmer).

Phosphorylation of the peptide substrate (50 μM) was terminated by the addition of 10% orthophosphoric acid, and aliquots were spotted onto phosphocellulose filters (Whatmann P81). Filters were washed with 1% orthophosphoric acid three times and dried before counting in scintillation counter.

### Molecular docking

CK2 protein structures were modeled and ligand binding studies were carried out using SwissDock (http://www.swissdock.ch/) web service developed by the Molecular Modeling group of the Swiss Institute of Bioinformatics (Lausanne, Switzerland). The modeled and docked structures were analyzed with UCSF Chimera 1.12rc software. The binding site of the structures was identified using the crystal structure of CK2α (PDB code 1PJK), CK2α′ (PDB code 3OFM), and holoenzyme CK2α_2_β_2_ (1JWH). The docked structures showed binding energy in the range of − 7.49 to − 8.48 kcal/mol.

## Results and discussion

**Fig. 1:**
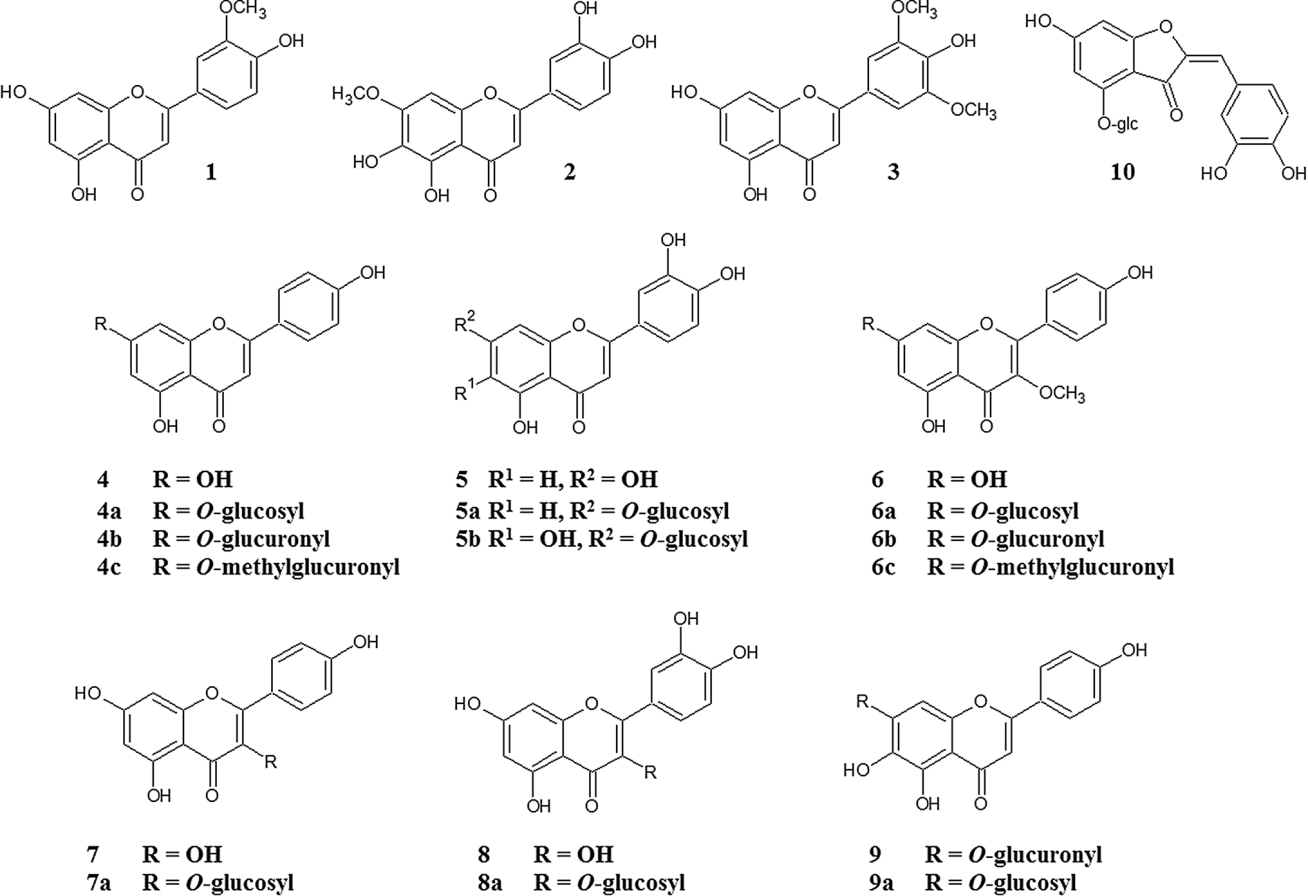
Structures of flavonoids tested in this study

**Fig. 2:**
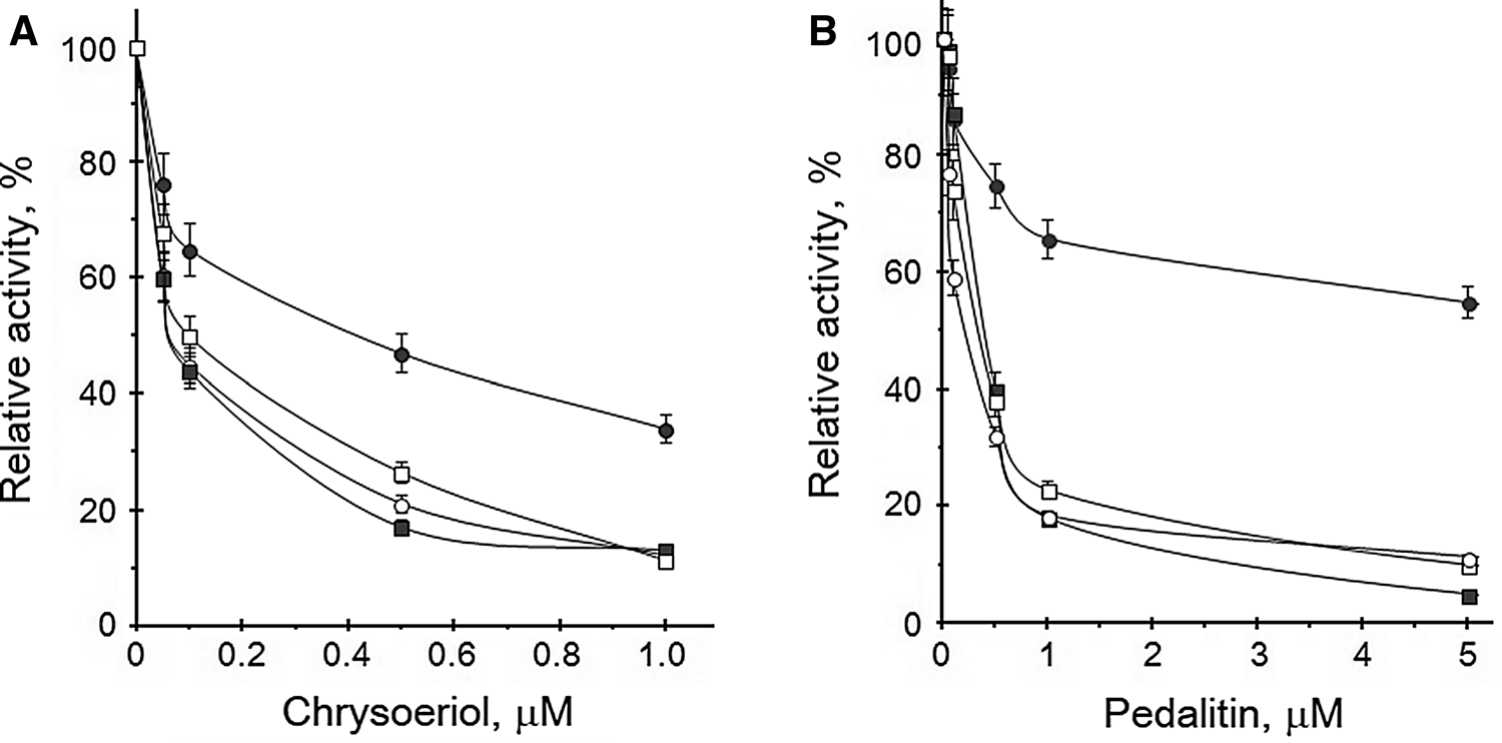
Inhibition of different CK2 isoforms. Comparison of chrysoeriol (**a**) and pedalitin (**b**) and their influence on CK2α (black circle), CK2α′ (black square), CK2α_2_β_2_ (white circle), and CK2α′_2_β_2_ (white square) phosphorylating activity. The experiment was carried out using 10 μM P2B as protein substrate and 20 μM ATP as phospho-donor. The results are the means of three independent experiments

In previous studies, we had described the inhibitory effect of different substances like halogenated *1H*-benzimidazoles and flavonoid compounds towards human catalytic subunits CK2α and CK2α′ [20, 23]. In our present study, we examined twenty-one flavonoid compounds (Fig. 1) for their potential influence on human protein kinase CK2 holoenzymes. We had already investigated those natural occurring compounds as inhibitors of free CK2 catalytic subunits. The obtained results showed differences between both subunits as well as between the used protein substrates, the acidic ribosomal protein P2B and the synthetic peptide RRRADDSDDDDD [23]. Therefore, we were wondering if similar results will be detected using the derived human holoenzymes CK2α_2_β_2_ and CK2α′_2_β_2_. A search in the literature revealed that apigenin (**4**), luteolin (**5**), kaempferol (**7**), and quercetin (**8**) were already tested on human CK2α_2_β_2_ and human CK2α′_2_β_2_ [21, 22]. Additionally, we included the compounds from our previous study which showed inhibitory potential towards CK2 catalytic subunits. Both recombinant holoenzymes were obtained by overexpression of CK2 subunits α, α′, and β in *E. coli*, mixing bacteria pellets expressing GST-CK2α and His-CK2β as well as GST-CK2α′ and His-CK2β and purified to homogeneity by affinity chromatography using glutathione-sepharose (GE Healthcare). The inhibitory effect was examined by increasing concentrations of the compound. The experiments were conducted using either P2B or the synthetic peptide as phosphoacceptor. Our obtained results are summarized in Table 1. First, we focused on those compounds exerting the strongest inhibitory effect towards both free catalytic subunits. Chrysoeriol (**1**), pedalitin (**2**), apigenin (**4**), luteolin (**5**), kaempferol (**7**), quercetin (**8**), and cernuoside (**10**) were tested towards both holoenzymes using P2B as substrate. A comparison of chrysoeriol (**1**) and pedalitin (**2**) in Fig. 2 illustrates the different potency of both flavonoids towards the free catalytic subunits and their derived holoenzymes. Surprisingly, IC_50_ values were lower using CK2α_2_β_2_ than for the CK2α′_2_β_2_ holoenzyme. In case of free catalytic subunits, the inhibitory effect was almost always stronger towards the CK2α′ subunit. Comparing the inhibition of the free CK2α′ subunit with IC_50_ values from 0.06 to 5.1 μM for chrysoeriol and cernuoside, respectively, with its holoenzyme (0.1–7.8 μM), the IC_50_ values were similar or higher for the holoenzyme. The difference between CK2α and the holoenzyme was significant, with IC_50_ values testing the free catalytic subunit of 0.42 μM for chrysoeriol and 9.8 μM for apigenin, but 0.06 μM for chrysoeriol and 1.5 μM for apigenin in case of the holoenzyme. Most potent inhibitor as in case for the free catalytic subunits is chrysoeriol (**1**) inhibiting all four molecular forms of CK2 with IC_50_ values below 0.42 μM. Interestingly, luteolin derivatives (**5a, b**) revealed opposite effect on CK2 than the parent compound. Whereas the inhibition is stronger comparing the holoenzyme and the respective free catalytic subunit (6.0 vs. 0.55 μM for CK2α and holoenzyme) in the presence of luteolin (**5**), both derivatives possess similar or even weaker activities towards the holoenzymes. Also, the strong inhibitory potential was lost in case of both luteolin derivatives (**5a**, **b**) on the CK2α′_2_β_2_ holoenzyme compared to luteolin (**5**) which was not the case for the free catalytic subunits. This decrease was even much stronger for the CK2α′_2_β_2_ holoenzyme with IC_50_ values about tenfold higher. Pedalitin (**2**), tricin (**3**), luteolin (**5**), kaempferol (**7**) are the only compounds inhibiting both holoenzymes better than the respective free catalytic subunits. Within the tested compounds, we found examples where inhibition of the holoenzyme was detected even in case where no effect was seen for the free catalytic subunit. Isokaempferide (**6**) influenced the CK2 activity of both holoenzymes with IC_50_ values of 17.5 and 12.1 μM. Its derivatives (**6a**–**c**) showed different effects with IC_50_ of 9.9 and 3.5 μM (compound **6b**) or without any inhibition (compounds **6a**, **c**). Otherwise, the same derivatives of apigenin (**4**), namely the 7-*O*-glucoside (**4a**), 7-*O*-glucuronide (**4b**), and 7-*O*-methylglucuronide (**4c**) inhibited with a similar extent (IC_50_ values between 11.2 and 16.5 μM) both holoenzymes but much less effective than the parent compound apigenin (**4**) inhibiting the CK2 activities to 50% at 1.5 and 2.5 μM.

**Table 1:** IC_50_ values (µM) of the tested compounds

Inhibitor	P2B				Synthetic peptide			
	CK2α^(a)^	CK2α′^(a)^	CK2α_2_β_2_	CK2α′_2_β_2_	CK2α^(a)^	CK2α′^(a)^	CK2α_2_β_2_	CK2α′_2_β_2_
**1**	0.42 ± 0.09	0.06 ± 0.01	0.06 ± 0.001	0.1 ± 0.008	0.25 ± 0.02	0.03 ± 0.007	0.08 ± 0.004	0.08 ± 0.07
**2**	7.0 ± 0.5	0.4 ± 0.05	0.2 ± 0.03	0.35 ± 0.03	0.4 ± 0.02	0.2 ± 0.03	0.2 ± 0.01	0.25 ± 0.05
**3**	21 ± 1.8	3.3 ± 0.5	1.55 ± 0.1	1.2 ± 0.1	11.2 ± 0.9	4.7 ± 0.4	7.2 ± 0.5	3.8 ± 0.4
**4**	9.8 ± 0.8	2.3 ± 0.1	1.5 ± 0.1	2.5 ± 0.2	3.9 ± 0.3	1.5 ± 0.2	0.8 ± 0.03	2.4 ± 0.2
**4a**	> 40	12.5 ± 0.9	11.2 ± 0.5	15.7 ± 1.2	> 40	> 40	12.1 ± 0.9	13.2 ± 1.1
**4b**	16.6 ± 1.4	0.4 ± 0.06	12.4 ± 0.6	16.5 ± 1.2	3.6 ± 0.2	5.4 ± 0.4	9.4 ± 0.6	22.8 ± 1.4
**4c**	> 40	3.3 ± 0.4	14.2 ± 0.6	12.5 ± 0.9	14.3 ± 0.8	24 ± 2.5	10.5 ± 0.8	5.6 ± 0.3
**5**	6.0 ± 0.7	1.0 ± 0.1	0.55 ± 0.07	0.65 ± 0.05	2.0 ± 0.1	0.75 ± 0.1	0.5 ± 0.02	1.0 ± 0.07
**5a**	17.5 ± 1.5	0.8 ± 0.1	31.0 ± 2.3	9.5 ± 0.7	0.1 ± 0.03	0.7 ± 0.1	9.0 ± 0.5	5.7 ± 0.06
**5b**	5.8 ± 0.5	0.35 ± 0.07	> 40	3.2 ± 0.4	7.0 ± 0.4	0.4 ± 0.05	6.2 ± 0.3	3.6 ± 0.04
**6**	> 40	13.6 ± 0.9	17.5 ± 1.4	12.1 ± 1.1	11.2 ± 0.9	13.7 ± 1.1	7.7 ± 0.4	10.2 ± 0.7
**6a**	> 40	> 40	> 40	> 40	> 40	> 40	> 40	> 40
**6b**	> 40	3.0 ± 0.2	9.9 ± 0.8	3.5 ± 0.1	> 40	7.7 ± 0.8	5.0 ± 0.2	5.3 ± 0.3
**6c**	> 40	> 40	> 40	> 40	17 ± 1.8	> 40	> 40	> 40
**7**	4.8 ± 0.4	1.0 ± 0.05	0.35 ± 0.04	0.35 ± 0.02	0.7 ± 0.1	0.2 ± 0.02	0.3 ± 0.02	0.3 ± 0.04
**7a**	> 40	> 40	> 40	> 40	> 40	> 40	> 40	> 40
**8**	1.8 ± 0.2	0.2 ± 0.007	0.25 ± 0.03	0.25 ± 0.02	0.5 ± 0.05	0.4 ± 0.03	0.5 ± 0.03	1.0 ± 0.08
**8a**	> 40	19.2 ± 1.5	24.5 ± 1.7	31.9 ± 2.6	3.7 ± 0.4	> 40	> 40	> 40
**9**	33 ± 2.8	4.3 ± 0.3	> 40	> 40	> 40	4.1 ± 0.5	19.1 ± 1.7	9.7 ± 0.8
**9a**	> 40	3.8 ± 0.2	0.4 ± 0.06	6.3 ± 0.5	3.0 ± 0.4	4.4 ± 0.5	5.4 ± 0.3	3.2 ± 0.2
**10**	5.8 ± 0.4	5.1 ± 0.3	3.7 ± 0.3	7.8 ± 0.7	5.3 ± 0.5	4.0 ± 0.3	5.1 ± 0.3	9.6 ± 0.8

Experiments were performed using yeast ribosomal protein P2B or synthetic peptide as substrate and in the presence of 20 µM ATP

The results represent the means of three independent experiments

^(a)^Data from ref. [23]

The glucosylation at position C-3 or C-7 dramatically lowered the inhibitory potency in case of derivatives of apigenin (**4a**), luteolin (**5a**), isokaempferide (**6a**), kaempferol (**7a**), and quercetin (**8a**). The decrease in the potential of these compounds lies between sixfold up to over 100-fold for apigenin 7-*O*-glucoside and quercetin 3-*O*-glucoside, respectively. Noteworthy compounds **6a** and **7a** did not show any effect on the CK2 activities.

Scutellarin (**9**) was shown to exhibit very weak potential towards CK2α but good inhibition in case of CK2α′. Similar effect was detected for scutellarein 7-*O*-glucoside (**9a**) which completely failed in case of CK2α. Interestingly, compound **9a** possesses very good inhibitory potential towards CK2α_2_β_2_ with an IC_50_ of 0.4 µM, but weaker potential towards CK2α′_2_β_2_ than on CK2α′ (Table 1).

**Fig. 3:**
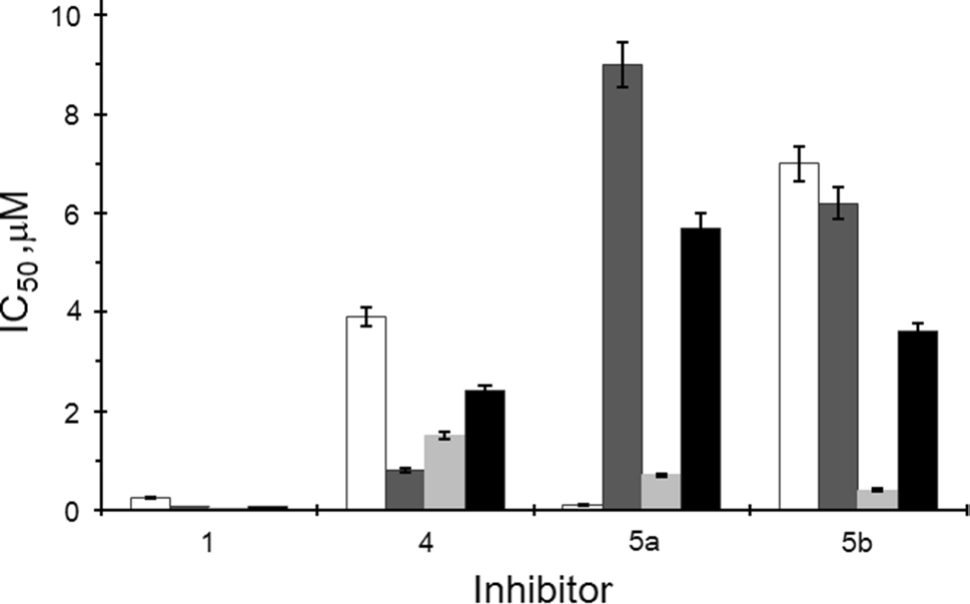
Comparison of compounds chrysoeriol (**1**), apigenin (**4**), luteolin 7-*O*-glucoside (**5a**), and 6-hydroxyluteolin 7-*O*-glucoside (**5b**) towards their inhibitory potential on CK2α (white), CK2α_2_β_2_ (dark gray), CK2α′ (light grey), and CK2α′_2_β_2_ (black) phosphorylation. The experiments were carried out in the presence of 50 μM of the synthetic peptide and in the presence of 20 μM ATP. The results are the means of three independent experiments

In most publications instead of a full protein substrate, like P2B, a synthetic peptide is used as phosphoacceptor containing the typical recognition site for protein kinase CK2. In most cases, CK2α_2_β_2_ was better inhibited than CK2α. As illustrated in Fig. 3 comparing flavonoid compounds towards the their influences on different CK2 isoforms revealed diverse results. Whereas mainly CK2α is less inhibited than CK2α′ in case of luteolin derivative **5a** the opposite is the fact. Similar behavior was detected comparing the IC_50_ values of CK2α and CK2α_2_β_2_. In most cases, the holoenzyme was stronger inhibited than the free catalytic subunit, but compound **5a** is one of the few exceptions. In general, the CK2α′ subunit is more sensible towards the tested flavonoid compounds than the derived holoenzyme. Quercetin (**8**) and cernuoside (**10**) possess similar inhibitory potential towards CK2α and its holoenzyme. Apigenin 7-*O*-glucuronide (**4b**) and luteolin 7-*O*-glucoside (**5a**) were less active on CK2α_2_β_2_ than towards CK2α but still had a moderate inhibitory effect with IC_50_ values of 9.4 and 9.0 µM. The *O*-glucoside derivatives of isokaempferide (**6a**), kaempferol (**7a**), and quercetin (**8a**) as well as isokaempferide 7-*O*-methylglucuronide (**6c**) were completely ineffective towards both holoenzymes similar like towards the free catalytic subunit with exception of **6c** and quercetin 3-*O*-glucoside (**8a**) with IC_50_ values of 17 and 3.7 µM towards CK2α, respectively (Table 1). Surprisingly, apigenin 7-*O*-glucoside (**4a**) showed similar inhibitory effect towards both holoenzymes, whereas it was inactive on free catalytic subunits. There are several compounds inhibiting CK2α′ and its holoenzyme in the same range, like pedalitin (**2**), tricin (**3**), apigenin (**4**), luteolin (**5**), isokaempferide (**6**), kaempferol (**7**). Other compounds, like chrysoeriol (**1**), quercetin (**8**), scutellarin (**9**), and cernuoside (**10**) showed a 2.5-fold stronger influence on CK2α′ than on the derived holoenzyme (Table 1).

**Fig. 4:**
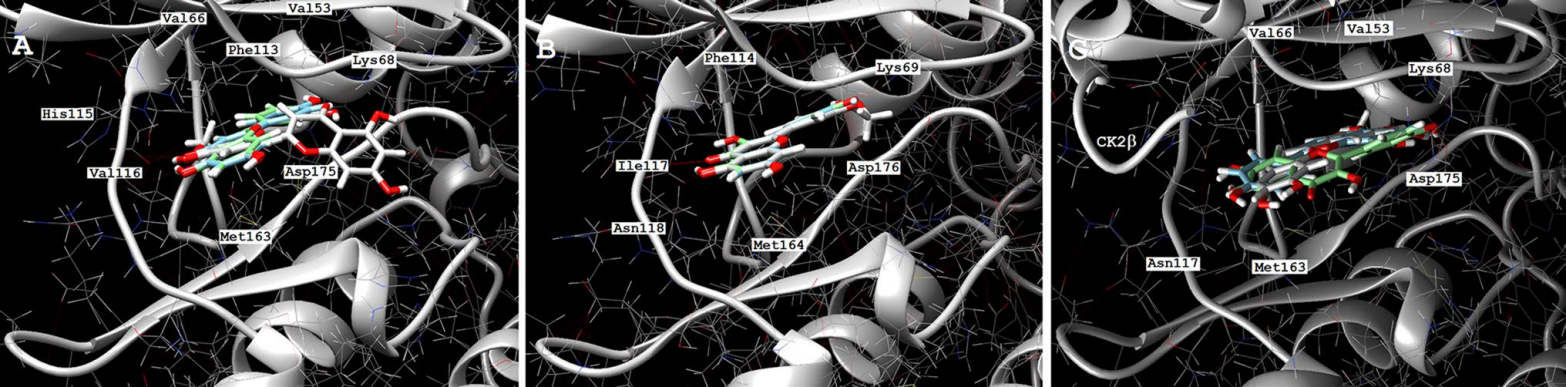
Comparison of the binding modes of chrysoeriol (white), apigenin (blue), and quercetin (green) to CK2α (**a**), CK2α′ (**b**), and CK2α_2_β_2_ (**c**) obtained with molecular docking. The docking was provided using the Swissdock web server and analysis was done with UCSF Chimera 1.12rc software. (Color figure online)

As already described by others [19, 22] and in our former work [23] flavonoids are strictly ATP-competitive inhibitors. Therefore, Lolli et al. tested some flavonoid compounds and their inhibitory efficiency against two CK2α mutants, Val66Ala and Ile174Ala [22]. Interestingly, the IC_50_ values of quercetin, luteolin, and apigenin were only marginally affected by both mutations. Therefore, we were interested to investigate the binding mode of three inhibitors, namely, chrysoeriol (**1**), apigenin (**4**), and quercetin (**8**) with the three available CK2 isoform structures (Fig. 4). The three compounds differs in their amount of hydroxyl groups and in case of chrysoeriol a methoxyl group at position 3′. Apigenin and quercetin, the latter possesses two additional hydroxyl groups at position 3 and 3′ compared to apigenin, bind in a similar manner to each CK2 isoform. A remarkable difference was seen when chrysoeriol was docked to the CK2α structure. As shown in Fig. 4a chrysoeriol binds in a 90° angle compared to the other both inhibitors to the ATP-binding site of the enzyme. By this way, the pocket is more blocked by chrysoeriol which could be the reason for its higher inhibitory potential. Also noteworthy is the slightly different position of chrysoeriol bound to the CK2α holoenzyme. Anyway, there is almost no difference detectable in the distance between inhibitor and amino acid chains ranging between 2.2 and 2.4 Å. Comparing the binding energy, we observed lower energy in case of the CK2α′/chrysoeriol complex than chrysoeriol complexed with CK2α and its holoenzyme. This is consistent with the results from the enzymatic studies where the inhibition is stronger for CK2α′. The interaction between flavonoids and CK2 isoforms is mainly due to the formation of H-bonds with Lys68 (Lys69 in case of CK2α′), Val116 (Ile117 in case of CK2α′), and Asp175 (Asp176 in case of CK2α′).

The CK2 holoenzymes differ in their biochemical characteristics which could be the reason for various sensitivity towards inhibitors. The study of Olsen et al. described the similarities and differences between the CK2α- and CK2α′-derived holoenzymes [36]. Interestingly, the CK2α_2_β_2_ holoenzyme is more stable at 45 °C due to the increase of the α-helical character of the subunits. Analysis by gel filtration revealed that the CK2α′_2_β_2_ holoenzyme exists only as protomer, whereas the CK2α_2_β_2_ holoenzyme is able to aggregate to higher molecular mass forms. In a later publication, the same research groups showed that CK2α′ is able to form a trimer (CK2α′β_2_) instead of the typical tetrameric form [37].

## Conclusions

Protein kinase CK2 exists as several isoforms, namely CK2α, CK2α′, CK2α_2_β_2_, CK2α′_2_β_2_, whereas, the holoenzyme is the predominantly occurring form in the cell.

As reported in several publications, the CK2β subunit regulates the preference for the protein substrate and possesses influence towards the structure of the ATP/GTP-binding site [11, 38, 39]. These interactions might be responsible for the different potential of the tested flavonoids in this work since flavonoids are pure ATP-competitive. To resolve this question, the crystal structure of bound inhibitor to the free catalytic subunit and the holoenzyme has to be done. Unfortunately, up to now, there is no crystal structure available for the CK2α′_2_β_2_ holoenzyme.

Flavonoids are promising compounds in the search for new CK2 inhibitors and in further future as potential anti-cancer drugs. There are numerous reports about their cytotoxic effect on cancer cell lines in vitro or chemopreventive possibilities as components of daily diet [40].
